# Evaluating the Impact of a Two-Stage Multivariate Data Cleansing Approach to Improve to the Performance of Machine Learning Classifiers: A Case Study in Human Activity Recognition

**DOI:** 10.3390/s20071858

**Published:** 2020-03-27

**Authors:** Dionicio Neira-Rodado, Chris Nugent, Ian Cleland, Javier Velasquez, Amelec Viloria

**Affiliations:** 1 Department of Industrial Agroindustrial and Operations Management GIAO, Universidad de la Costa, Barranquilla 080002, Colombia; jvelasqu3@cuc.edu.co (J.V.); aviloria7@cuc.edu.co (A.V.); 2 School of Computing, Ulster University, Shore Road, Newtownabbey, County Antrim BT37 0QB, Northern Ireland, UK; cd.nugent@ulster.ac.uk (C.N.); i.cleland@ulster.ac.uk (I.C.)

**Keywords:** multivariate analysis, HAR, machine learning, dataset quality

## Abstract

Human activity recognition (HAR) is a popular field of study. The outcomes of the projects in this area have the potential to impact on the quality of life of people with conditions such as dementia. HAR is focused primarily on applying machine learning classifiers on data from low level sensors such as accelerometers. The performance of these classifiers can be improved through an adequate training process. In order to improve the training process, multivariate outlier detection was used in order to improve the quality of data in the training set and, subsequently, performance of the classifier. The impact of the technique was evaluated with KNN and random forest (RF) classifiers. In the case of KNN, the performance of the classifier was improved from 55.9% to 63.59%.

## 1. Introduction

Criteria for the rejection of extreme observations as significant outliers in a one-dimensional normal distribution with unknown parameters has been widely studied [1,2,3]. In univariate analysis, suspicious observations which are declared as outliers are obvious by simple inspection. Additionally, there are simple statistical univariate procedures that can be used to declare an observation as an outlier [4]. The use of robust methods for detecting outliers in multivariate cases is also relevant and essential, considering the vast amounts of data being generated by pervasive computing and internet of things environments. An example of the inherent difficulty of the multivariate analysis is the interrelated character of outlier effects on location, scale, and orientation [2]. In real life situations, researchers are often faced with large amounts of data, and if they are not properly statistically screened, ensuing analyses may lead to the wrong conclusions.

On the other hand, the increasing use of information and communication technologies (ICT) devices and pervasive availability of the internet, allow users to monitor their heart rate, steps, distance travelled, and other variables important to monitor their health condition. In this case, to perform a faithful tracing of a user’s health condition, it is necessary to identify abnormal observations by considering these variables simultaneously in order to give to the user a trustable alert of his condition. One common method used to detect these abnormal observations in healthcare sector is the multivariate analysis based on Hotelling’s T^2^ [5,6,7].

The aging population is related with an increase of conditions such as stroke and dementia [8,9]. Because of these conditions, patients suffer a deterioration in both physical and mental conditions, making them dependent and vulnerable in the realization of their activities of daily living (ADL), making it necessary in most cases that patients require assistance. Considering these situations, and in order to reduce the negative impact dementia has in the quality of life of the elderly, the use of assisted living systems has arose as a very promising way to achieve a better quality of life of dementia patients [10,11]. These systems aim for achieving more independence and autonomy for elders, who live alone and at the same time enhance their quality of life. As a result, the cost for society and public health systems could be reduced [8,12]. 

Assisted living systems (ALS) rely on human activity recognition (HAR) as a key component of functioning [13]. HAR applications to ALS, range from promoting physical activity to monitoring of long-term chronic conditions [13]. HAR is concerned with the ability to automatically recognize and interpret human activity using computational methods, which remains a highly complex task [13,14]. The recognition of activities or actions can be determined through the interpretation of human body motion or gestures, captured via sensors, such as body-worn accelerometers. This kind of application can be found in previous works such as [15]. In this work, a new deep convolutional neural network structure is proposed to improve reconstruction and reduce the online prediction time of the performance of a 7 DoF anthropomorphic robot arm (LWR4 +, KUKA, Germany). While in [16], a new fast and robust deep convolutional neural network structure for the recognition of human activity (HAR) using a smartphone is proposed. Nevertheless, it is important to point out that HAR still requires significant improvement [17]. Considering that HAR is based on machine learning classifiers, it is necessary to reduce noise (outlier elimination) from training data, in order to obtain accurate activity predictions.

In this work, noise elimination is undertaken on multivariate outlier detection based on the calculation of the Mahalanobis distance. This is a novel approach to eliminate outliers in HAR datasets in order to improve the quality of the data. Although Mahalanobis distance is a well-known outlier detection technique, as far as we know it has not been implemented in HAR processes to improve the performance of KNN and/or random forest classifiers. Additionally, this research addressed the outlier detection in a two-step approach, first to clean the data within each user and then the overall aggregate data. The remainder of the paper is structured as follows. Section 2 provides a review of the literature in data cleansing and the effects data quality can have on classification. Following this, Section 3 describes the dataset collected and utilized in addition to the methodology used within the activity recognition process. The study case is presented in Section 4. Section 5 contains the analysis of the results obtained with the implementation of the proposed methodology to the study case. Finally, Section 6 shows some conclusions derived from the present study. 

## 2. Literature Review

### 2.1. Human Activity Recognition

HAR is a key element in many areas such as ambient assistive living, connected health and pervasive computing [16]. HAR becomes very useful in order to manage and prevent chronic diseases, through the surveillance of daily activities of elderly residents [14]. In this sense, due to the exponential development of ICT and internet of things (IoT), it has been possible the creation of smart homes. These smart homes are home environments, in which there is a full interaction between sensors within the house and user wearable sensors that make it possible to monitor their health and wellbeing by tracking ADLs through HAR techniques [14]. HAR is generally split into two types. The first type is the sensor-based HAR and the second type is vision-based HAR. Of these two, the first one has been more developed due to the recent progress in sensors and IoT technologies [17,18]. Regarding sensor-based HAR, a commonly used HAR wearable sensor is the accelerometer. In general, wearable sensors, such as the accelerometer, are frequently used in HAR due to the easiness for real-time implementation, the fact of not being location dependent, and their low cost [19,20]. Additionally, this type of HAR device is considered suitable for monitoring activities such as walking, running, standing, sitting, and ascending stairs [16]. These activities are of the same type of the ones performed in this study case.

In HAR, the selection of the sensor type is as important as managing the huge amount of data, collected by the sensor. In this sense, analytic phases—such as pre-processing, data segmentation, extraction of discriminative features, and finally classification of activity details—must be properly executed in order to obtain a good classifier performance [21]. Pre-processing deals with the detection and management of outliers and missing data [21]. The segmentation step refers to the process of grouping data in a window size in order to extract useful features. The size of the window depends on the type of activities to which the HAR process will be carried out. In-sensor data segmentation is very common to use methods such as ranging from sliding windows, events, or energy-based activities [21]. Once data have been segmented, different techniques are used in order to identify which of the extracted features are relevant, in order to minimize classification errors and reduce computation time. It is also important to point out that selected features can be split into statistical and structural ones [21,22]. Features—such as mean, median, and range among others—are known as statistical features. These features correspond to descriptive statistical analysis within the time window of sensor data. On the other hand, structural features—such as entropy, energy, and others—are extracted from the relationships among the different sensor data. 

Finally, machine learning and/or pattern recognition methods are used in order to be able to identify which activities have been performed if the sensor gathers some particular measures. This phase is known as the activity recognition and classification phase [21]. There are a great number of useful machine learning techniques for this purpose. These include the support vector machine (SVM), K-nearest neighbour (KNN), and others [13,21]. Recently, in order to improve the ability to perform a better activity recognition, many studies have used information gathered with different sensors at the same time. This approach has allowed researchers to gain robustness, and reliability, in the process of recognizing human activities [23]. 

According to Domingos [24], feature extraction is the most important stage in the HAR process. Therefore, extensive works have been conducted in order to improve HAR process through the extraction of expert-driven features [22]. Nevertheless, expert-driven features extraction methods depend on the knowledge of the experts and applicability of the feature vectors in the problem domains. Furthermore, hand-engineered features cannot represent the outgoing characteristics of complex activities and involve time-consuming feature selection techniques to select the optimal features [25]. Considering this difficulty, deep learning has gained a lot of focus due to its ability to learn features from data [26]. In this sense, convolutional neural networks (CNN) were initially used to tackle image recognition problems, in which they proved to be effective for such tasks. Applications can be found in [27,28,29,30,31] and include photography recognition and videos recognition. The ability CNN have in the identification of local correlations, is what makes them adequate for image processing. Therefore, in order to take advantage of CNN’s properties, in sensor-based HAR applications, many researchers transform motion signals into images. The transformation process of sensor-based signals into images, can be accomplished by different ways. One possibility is reordering signal values into an image. In other cases, researchers construct the corresponding signal spectrogram. The last alternative is to calculate features from a signal window and then put them into a graph [32]. Nevertheless, researchers have found that the performance obtained in the classifiers when automatically generating features via CNN is similar to the performance obtained with hand-crafted features [33]. This makes it necessary to evaluate in each case the convenience of automatically generate features through CNN, or work with hand crafted features that have been developed across the years. The key factor is the evaluation of the impact that each action taken in the preprocessing step, segmentation step, feature calculation and selection step, and the model selection step, have on the performance metrics—such as accuracy, F-measure, and others. In this sense, it is also important for the researcher to have a clear idea of which of these measures is most important according the particular application being analyzed.

Within the field of data-driven approaches to HAR, data quality is a significant consideration. In this sense, data cleansing represents a relevant procedure to improve the quality of datasets and to improve the outcomes of the classifiers. Data cleansing can be defined as the process of removing errors or inconsistencies such as noise and/or outliers from a collection of data [34]. The presence of noise and outliers in data is an important issue to address as these can have a substantial influence on the results produced by data driven techniques, according to Gorunescu [35]. Nevertheless, Han et al. [36] state the border between normal and abnormal (noise/outlier) data is often unclear, where a large “gray area” may exist. Noise can be presented at a class or attribute level in supervised learning, with previous effort made by [37] to evaluate their impacts on classification accuracy on 17 datasets, collected from various domains. Various levels of noise were manually applied to each dataset, with conclusions stating that as noise levels increase, classifier performance decreases further, and that class noise is usually more harmful than attribute noise. Further to this, [38] compared numerous classifiers to evaluate how well they performed with noisy data, with conclusions stating that performance and robustness to noisy data varied substantially between various algorithms. The random forest classifier proved to be most resilient to noise, followed by KNN. Other studies considering data quality included investigations made by [37,39,40]. In [39], a fuzzy relevance vector machine (FRVM) was used to learn from unbalanced data and noise, with experiments demonstrating reasonable and more robust performance than a regular RVM. Then, inclusion of fuzzy mathematics helped to make the RVM most robust. In [37] and [40] respectively, noise impact was analyzed through different experiments with datasets available in the literature. In this sense, both studies concluded that class noise (wrong labeled instances in the dataset) affects the learning process of the classifiers and explore different ways to detect this problem and deal with it. On the other hand, both studies show that attribute noise (noise within the instances data, such as outliers, and missing data) represents a more difficult challenge, considering that each observation may contribute to the learning process of the classifier and the elimination or replacement of a particular data in the instance may affect the performance of the classifier. Therefore, it is relevant to look for different ways to deal with attribute noise. In this case, the proposed approach deals with the problem of attribute noise with multivariate outlier detection. 

### 2.2. Multivariate Outlier Detection

Unlike univariate outlier management, managing multivariate outliers is a difficult task. Its difficulty lies on the fact that multivariate outlier detection must consider the correlation between the different variables involved in the analysis. This situation may mean some observations that were considered within the acceptable variation range of an individual variable to then become considered as outliers when this correlation is considered [41]. In the process of multivariate outlier detection, researchers may have to deal with two situations. The first situation or effect is known as the masking effect. In the masking effect an outlier shadows a second outlier. This means that the second outlier is considered as such, only by itself, but not in the presence of the other outlier. The second effect is known as the swamping effect. In the swamping effect, a second outlier is considered an outlier only in the presence of the first outlier.

These two effects may affect type II and type I errors values respectively. Type I error refers to the false positives and type II error refers to the false negatives. Many researchers have studied the consequences of the presence of both masking and swamping effects and have also proposed methods to deal with them. Related studies can be found in Iglewics and Martinez [42], and Becker and Gather [43].

#### 2.2.1. Robust Distance Methods

In order to detect outliers, robust based methods are the most commonly used by researchers managing multivariate datasets. These methods are usually based on local distance measures and are capable of handling large databases [41]:

- The Mahalanobis robust distance:

The Mahalanobis distance (MD) is a well-known method for multivariate outlier detection which depends on estimated parameters of the multivariate normal distribution. In the calculation of the MD, *n* observations are taken from a *p*-dimensional dataset. In this case, *p* represents the number of variables considered in the outlier detection procedure. In this process the mean of each variable within *p*, is organized in a vector denoted as ***μ*** and known as the mean vector. Additionally, the sample covariance matrix is constructed and denoted as ***V***. The Mahalanobis distance for each multivariate data point *i, i* = 1, …, *n*, (denoted by $M_{i}$) is calculated with the equation

(1) $$M_{i}={\left[\overset{n}{\underset{i=1}{\sum }}{\left(X_{i}-\mu \right)}^{T}\;V^{-1}\;\left(X_{i}-\mu \right)\right]}^{1/2}$$

If the MD is greater than a threshold value, the observation is labeled as an outlier. The threshold value corresponds to the chi square distribution with α significance level and *p* degrees of freedom. Depending on the situation and the difficulty to fulfill the multivariate normality assumption, some researchers have proposed the use of a vector of medians instead of a vector of means [44]. Penny and Jolliffe [45] presented a modification of the procedure presented in [44]. They proposed the construction of a covariance matrix, based on weighted observations according to their distance from the center. Other robust estimators—such as M-estimator (M stands for maximum likelihood type) and S-estimator (scale estimator), among others—are considered as effective ways to detect multivariate outliers [41,46,47].

#### 2.2.2. Non-Traditional Methods Based on Robust PCA (Principal Component Analysis)

Many researchers have proposed alternative methods to deal with multivariate outlier detection. These methods try to overcome the implicit difficulty of robust distance methods requiring a subset of outlier-free data from which the mean vector and the covariance matrix can be constructed. Unfortunately, there is no existing method that can find an outlier-free subset with 100% certainty. The two most relevant of these PCA methods are the method for outlier identification in high dimensions which is based on the determination of semi robust principal components and the method based on functional approach. In this case, outliers are labeled using functional equivalents of boxplots and bagplots [41]. Applications and adaptations of these methods can be found in Hyndman and Shang [48], Croux and RuizGazen [49], and Rousseeuw et al. [50].

In Table 1, it can be observed some recent and relevant research related with outlying detection. The approach, scope as well as relevant issues of the research are depicted. It can be concluded that for high dimensional data Mahalanobis distance is considered as a recommended technique for data cleansing. Different approaches have been proposed but many of them are tested in small dimensional data, or are not tested with RF, nor KNN classifiers.

## 3. Methodology

This paper aims to present an approach for the identification of multivariate outliers. The dataset utilized in the current study was collected by students enrolled in the Pervasive Computing in Healthcare module at Ulster University using a wearable triaxial accelerometer, placed on the dominant wrist [11]. The students were each assigned to an AR scenario and perform three activities within the scenario. Then, they had to collect, process, and classify data as part of the module assessment. In total, there were 6 scenarios and 18 activities recorded amongst the cohort. On Table 2, can be seen the 6 scenarios and the 18 activities recorded during the experiment.

Data was collected with sample rate of 51.2 Hz. Then data were windowed, considering time windowing, as is a common approach with accelerometers where sensor data is sampled at a sustained rate. Nevertheless, there is still no clear consensus, regarding the window size that should be implemented. If the window size is too small, there may not be enough relevant activity information. On the other hand, if the window size is too broad, the measurements made, may have too few variations to result in appropriate decisions for activity classification [15]. A non-overlapping window size of 4 s was considered appropriate [58] as it has been observed that energetic activities such as walking, jogging, and running can be optimally detected between 1 s and 3.25 s, while more complex activities may require longer time windows [59]. Data were collected with the use of the Shimmer wireless sensors platform. Before the start of the data acquisition process the device was calibrated following the manufacturer guidelines. The Shimmer device was placed in the dominant wrist of each student, and acceleration data across *x, y*, and *z* axis were gathered (see [11]). As previous studies have shown, placing the sensor in the wrist provides trustable information to be used in HAR processes [59]. For data cleansing and windowing was used R^®^ (3.6.2 version), and the classifiers and its performances measures were obtained with Weka^®^ (3.8.3 version).

### 3.1. Feature Extraction

A compilation of standard time and energy domain features were extracted from the windowed data to obtain relevant information and to represent the characteristics of various activity signals. The features included the mean, natural logarithm, exponential, maximum, minimum, standard deviation, variance, root mean square (RMS), signal magnitude area (*SMA*), range, and median for the *x*, *y*, *z* axis and signal magnitude vector (*SMV*), and the cross correlation for each axis, as it has been suggested that these features are suitable for activity recognition [13,60]. In total, 51 features were extracted. The features used are presented in Table 3.

The statistical features mentioned are common due to their simplistic nature and significant performance across a variety of activity recognition problems [13,60]. The maximum, minimum, and range features can assist in differentiating between activities that contain movements comprised of different ranges. Signal magnitude area (SMA) has proved beneficial when employing triaxial accelerometers for activity recognition as it can suitably differentiate between static and dynamic activities. SMV signals are independent of the orientation of the sensor and are consequently valuable to include as the dataset contains a large number of participants, each placing the sensor on their dominant wrist.

### 3.2. Process Stages

**Figure 1:**
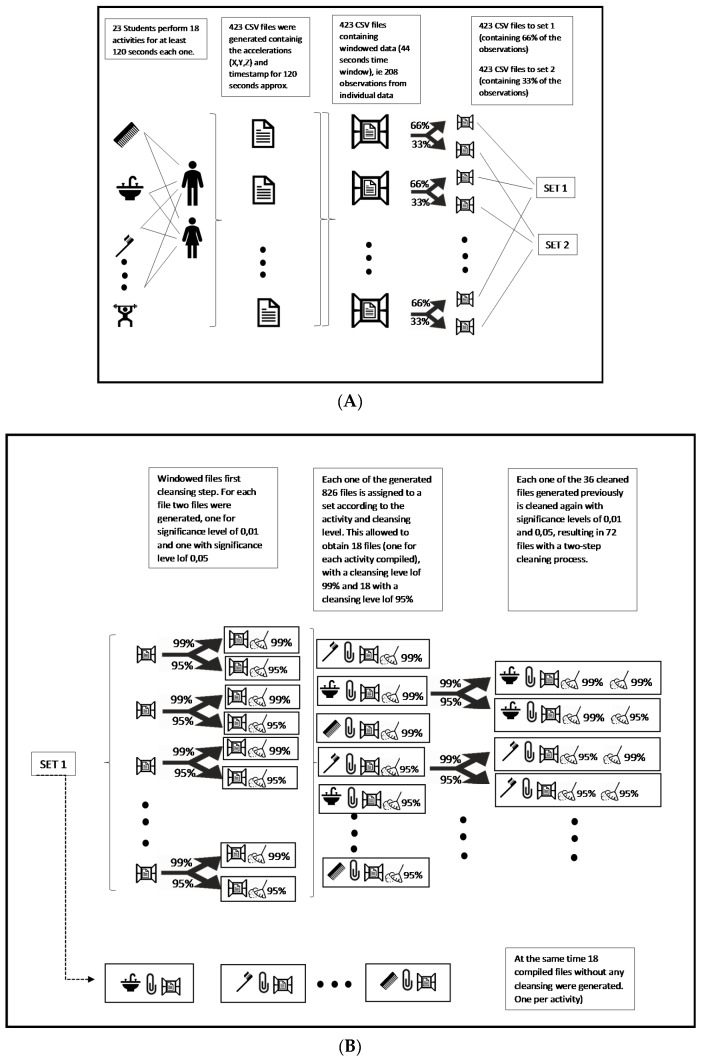

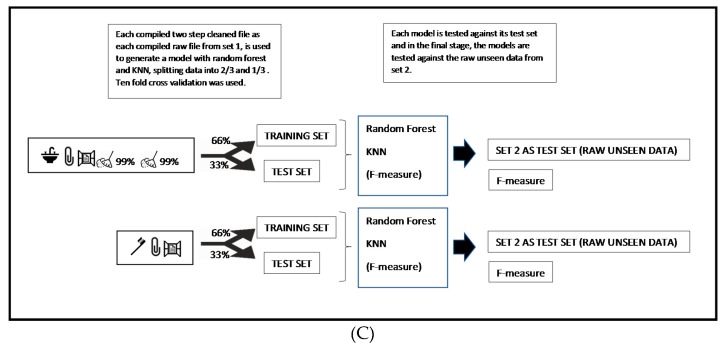
(**A**) Proposed approach representation. This representation corresponds to stage 2 of the process. (**B**) Proposed approach representation. This diagram corresponds to the stage 3 of the process, in particular to phases 1 to 5. (**C**) Proposed approach representation. This diagram corresponds to the stage 3 of the process, phases 6 to 8.

The implemented procedure adopted in the current work is described below. The importance of the use of this technique lies on the fact that this technique considers the correlation of the data in order to detect outliers. This approach has some relationship on KNN principle and is suitable considering that each participant performed each activity independently for 2 min and recorded his observations in a separated file. Other approaches to detect outliers in HAR, use clustering or other machine learning techniques to detect outliers. This kind of implementations are described in [55,58]. The proposed approach is described below:

1. Stage 1: In this first stage, an evaluation of different classifiers in order to determine the two most suitable for the HAR with the collected data. The output confirmed the fact that random forest and KNN are the most resilient classifier when working with noisy data [38]. This stage was useful only to determine the classifiers that were going to be used to evaluate the proposed data cleansing approach (see Section 4.1).
2. Stage 2: Once it was clear that the best classifiers were KNN and random forest, the generated database is managed and split in order to obtain two sets. Set 1 containing two-thirds of the observations. This set will be used for data cleansing approach evaluation, model creation, and evaluation. This means that from this set, the process of training and evaluating using the 10-fold cross validation will be performed. Set 2 will remain with raw data in order to test the classifiers against unseen data. This stage is depicted on Figure 1A.
3. Stage 3: Once the two sets are obtained in stage 2, set 1 was used to train the classifiers with noisy data. The purpose of this, is to evaluate the performance measures of the classifiers when trained with noisy data and compared it with the performance when trained with cleaned data. In this case, clean data refers to the resulting set once the proposed cleansing approach is implemented to this set 1. The detailed process in this stage is described in phases 1 to 8 and on Figure 1B (Phases 1 to 5) and on Figure 1C (phases 6 to 8).
  The phases carried out in this stage are:
  1. Phase 1: Feature calculation and windowing of the data.
  2. Phase 2: Multivariate outlier detection in the particular windowed data of each student. This outlier detection was made with the two proposed levels of 95% and 99% confidence levels. This means working with alpha levels of 0.01 and 0.05. These two levels are the most widely used for this kind of tests [61]. To perform this multivariate outlier analysis, the MD was computed using the mean of windowed data from acceleration across *x*, *y*, and *z*-axes.
  3. Phase 3: Compilation of the cleaned data in 18 files (one for each activity). Phase 3A: On the other hand, the raw files were compiled in 18 raw files too. One for each activity.
  4. Phase 4: Multivariate outlier detection of the compiled data. This outlier detection was made with 95% and 99% confidence levels.
  5. Phase 5: Compilation of the cleaned 18 files. Considering the previous cleaning processes and the different cleansing levels, up to this point four files were obtained. One with 95% confidence level in step 1 and 99% confidence level in step 2, one with 95–95%, one with 99–95%, and the fourth file with 99–99%.
    Phase 5A: Compilation of the 18 raw files in one file.
  6. Phase 6: Load the compiled files to Weka^®^.
  7. Phase 7: Apply a supervised attribute selection technique D1–N5 from Weka^®^ (3.8.3 version).
  8. Phase 8: Create the model (KNN or random forest). The model was created using a 10-fold cross validation with a two-thirds training and one-third testing purposes. All steps were performed with set 1. F-measure values were extracted for each model. Considering that the data were cleaned with different levels in step 1 (Phase 2) and step 2 (Phase 4), four models were created for KNN and four for RF.
    Phase 8A: A similar way to the described in Phase 8 was followed with the raw data. In this case, a model with KNN and a model with RF were generated.
4. Stage 4: Repeat stage 2 and stage 3 10 times. So, in the end, 100 models will be generated.
5. Stage 5: Analysis of results. This analysis will be performed once the approach is applied to the study case data.

## 4. Study Case

As it was mentioned before the proposed approach will be tested at the Pervasive Computing in Healthcare module at Ulster University. The Smart Environments Research Group at Ulster University is working to improve the performance of different classifiers for Human Activity Recognition. One of the branches of this research target, is the evaluation of different ways to clean data, in order to evaluate the impact of these actions in the classifier performance. Therefore, the first stage of the process was to determine through experimentation the best classifiers for the activities performed by the students. The school have previously tested manual data cleansing with no effect on the performance of the classifier when tested against raw unseen data. In order to tackle this situation, this new approach was proposed in order to evaluate the impact on the classifier’s performance.

The implementation of the different stages and steps described in the methodology to the study case are depicted below.

### 4.1. Stage 1

Different tests were initially performed in order to determine the best two classifiers for the constructed dataset, so it was possible to evaluate the impact of the proposed cleaning process. At the beginning KNN, SVM, random forest (RF), neural networks, and regression were taken in consideration for the study, due to the fact that are the most commonly used for this task [13,62,63]. The classifiers with the best outcomes were KNN and RF. Once this was determined, data cleansing was performed to the supplied data trying to improve the quality of the data in order to obtain a better performance of the classifiers. The original supplied data was formed by 423 files each one containing approximately 6000 observations of acceleration across *x*-, *y*-, and *z*-axis. The files can be grouped in 18 sets depending on the activity performed during data acquisition. The distribution of the data in the 18 activities can be found in Table 3.

**Figure 2:**
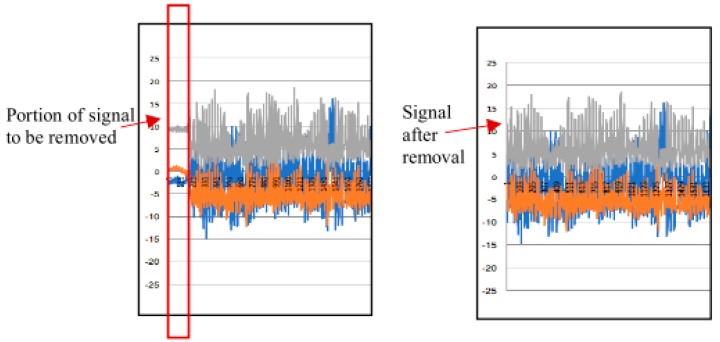
Signal manual cleansing. The values in the *y*-axis correspond to acceleration in m/s^2^. The *x*-axis shows the evolution of the observations. Each second, 51.2 acceleration data are gathered by the shimmer. This means the value of 100 in *x*-axis correspond to the time 1.95 s. The blue, orange, and grey lines represent the acceleration in *x*-, *y*- and *z*-axis.

The noisy data were used to evaluate the performance of a range of classifiers. This process was performed using the Weka^®^ software (3.8.3 version). Some manual cleansing was made prior the classification process. This manual cleansing focused on eliminating data at the end and at the beginning of the file in each one of the 423 files. This process was very helpful in order to eliminate data at the beginning of the observation of each activity for each person. The aim of this process was to take into account only the observations when the person was performing the activity correctly, i.e., eliminate the data of the learning curve (Figure 2). The remaining observations (after the manual cleansing) were grouped in 4-s time windows. The aim of the cleaning process is to identify corrupt (noisy) data that could be causing that the classifier does not learn properly from the training set. This inadequate learning process relates with the fact that the corrupt data make it difficult for the classifier to establish a relationship between a particular label with a particular set of observations. This classifier ‘confusion’ may generate that it incorrectly classifies some instances when tested against raw unseen data. This noisy data elimination process should provide better quality data so that the classifier has a more trustable training process (less corrupt data), and in having so, the classifier should have a better performance in identifying the activity that is being carried out. 

The data were cleaned and compiled using the software R (3.6.1 version). This first stage yielded the results as presented in Table 4 and Table 5. These results were obtained when working with the cleaned data and were improved in all the cases when compared with the case when the model was built with raw data. In each case, a 10-fold cross validation procedure was used. According to these results KNN and random forest were selected as the most suitable classifiers.

Additional tests were carried out at this stage trying to identify the effect on the performance of the classifiers when more instances were removed. These additional tests were carried out only with the selected classifiers (KNN and RF). The results are presented in Table 6.

Each case presented in Table 6 corresponds to a particular cleansing level. This cleansing level is associated with the removed instances. For example, case 1 corresponds to a cleansing level, in which 15.93% of the instances were removed, and case 5 corresponds to the raw data. In all these cases, the manual cleaning levels were made before splitting the data into the training set and the test set. Therefore, the impact on the F-measure is that the more removed instances the higher F-measure values obtained. Nevertheless, these results are not useful in real life, in which the classifier must deal with noisy data. Therefore, the challenge lies on cleaning the data to a point in which learning improves and helps the classifier face real life conditions (datasets containing noisy data).

At this stage, another important test was carried out. This test consisted of evaluating the impact of the number of features used for estimation. Results for KNN with 27 and 3 features are presented in Table 7. These tests were performed considering also, different cleansing levels. In the case of scenario 2, the data cleansing was stronger that in the case of scenario 1. These scenarios correspond to cleansing levels similar to case 3 and case 4 presented in Table 6.

In Table 7 is the value of F-measure for KNN classifier in particular working with 27 features and 3 features can be observed. The performance with 27 features is 30% better than when working with just 3 features.

### 4.2. Application of Mahalanobis Distance-Based Multivariate Data Cleansing (Stages 2, 3, and 4)

In stage 1, the purpose was to explore the data and select the most suitable classifier in addition to identify the impact of the number of features on the performance of the classifier. Now that random forest and KNN were selected as the most suitable classifiers, and the importance of having more features has been highlighted, the implementation of the stages 2, 3 and 4 can be followed. All the files were processed according to these stages. First, individual features were calculated. Then the files were windowed considering a 4 s time window. Then windowed features were calculated, such as mean, range, and standard deviation, among others. This processing was performed on each of the 423 files corresponding to the different persons and activities. For each case the data was split into two sets. The set 1 for training purposes amounts to 66% of the observations and the other 34% was used for testing purposes. This split was made 10 times, allowing 10 training sets and 10 test sets to be obtained for each one of the 423 files. The data splitting was performed randomly using R.

Considering that the process of data acquisition focused on 3 variables (acceleration across *x*-, *y*-, and *z*-axis) and knowing that each activity has a particular relation between the three values, the proposed approach aimed to identify the noisy data (outliers). This proposed approach is based on the application of multivariate outlier detection. This process consists of the calculation of the MD. As previously described, MD is like the Euclidean distance, however, it considers the fact that there are correlations between data (the three values of acceleration in this case). In the case that the correlation between the data is not considered in the process the outcome would be a sphere. The inclusion of correlation in the process yields a tridimensional oval. The shape of the oval depends on how strong the correlation between variables is. This procedure is more effective in outlier detection considering processes where the monitored data are correlated. The border of this oval is defined by the value of the chi square test at a desired significance level.

For each of the 423 files the MD was calculated, and data cleaning performed as a first step. This cleansing process was carried out considering a significance level of 0.05 and 0.01 with the chi square test. Once this cleansing was performed the data were compiled considering the activity. This yielded 18 files for training purposes cleaned with a significance level of 0.01 and 18 with a significance level of 0.05. Similarly, with the test data, 18 with a significance level of 0.01 and 18 files with a significance level of 0.05.

Considering that each one of these files contains observations from different participants, a second cleaning process was performed. The purpose of the first cleaning was to eliminate outliers within each participant performing each activity. This second cleaning process aims to eliminate outliers from the aggregation of participants performing the same activity. The aggregation is made using the previously cleaned data, forming one set with the significance level of 0.01 and a second set with the file cleaned with a significance level of 0.05. The outlier detection in these compiled sets was made considering also two significance levels (0.01 and 0.05). At the end there were 18 files with a cleaning process with a confidence level of 95% (significance level of 0.05) in the first step and 95% in the second. Another 18 with cleansing level of 95–99%, another 18 with 99–95% and 18 with 99–99%. All of these cleaned files yielded four compiled files for training and four for test purposes. It is also important to point out that there was a training set made of raw data, containing all the observations from the training set extracted from set 1. There will also be a test set compiled file with raw data. This test set corresponds to set 2, and its observations remain unseen for all the trained models in the process.

Considering that the selected classifiers were KNN and RF, and that this process was repeated 10 times, this yielded 40 cleaned training files for KNN and RF, 40 cleaned test files, 10 raw training files, and the corresponding 10 raw test files. The results of the F-measure for KNN, RF are presented for each repetition and cleaning levels in Table 8, Table 9 and Table 10.

The values of F-measures presented in Table 8 and Table 9 correspond to the values obtained when the model was trained with a training set with a particular level of cleansing and tested against the test set with the similar cleansing level. On the other hand, there were models of KNN and RF created with raw data and tested against raw unseen data. The values obtained for F-measure under these circumstances are presented in Table 10. 

Nevertheless, the models created with cleaned data were saved and tested against raw unseen data (set 2) in order to evaluate the impact of the cleaning process. The F-measure values are presented on Table 11. The purpose of this test was to evaluate the performance of the classifier in conditions similar to real life, in which there will be no cleansing process in the process of generating the data to predict the activity that is being carried out.

## 5. Analysis of Results

From Table 11, it can be inferred that the performance of the classifiers differs from the value obtained in Table 8 and Table 9. The difference in the performance lies on the fact that the models become overfitted, despite of the use of a 10-fold cross validation procedure. The values of F-measure of the 10 repetition were annotated and its mean and standard deviation were calculated. In order to perform statistical comparisons (*t*-tests) and verify if there are statistical significant differences between the classifiers and levels of cleansing, normality tests were executed with the 10 values obtained for each combination of cleansing level and classifier. This is important in order to guarantee the validity of the *t*-test. On Table 12, Table 13 and Table 14 it can be observed the mean and standard deviation of each classifier, and *p*-value for Anderson Darling normality test. The test was made considering a significance level of 0.01.

Considering that in all models *p*-value > 0.01, then normality assumption is valid, and *t*-tests can be carried out. The results of the *t*-tests between the different KNN models shown on Table 10 can be found on Table 15 below.

The results of the *t*-tests between the different RF models shown on Table 12 can be found on Table 16 below.

Similarly, considering that the data from Table 14 are normally distributed similar comparisons can be performed. Initially, it was important to determine if there is a significant difference in the performance within the different KNN models when tested against raw data. In order to determine this a one-way ANOVA (significance level of 0.01) was carried out. This ANOVA yielded a *p*-value = 0.99. This result implies that the cleaning level carried out with KNN has a similar effect, and it makes no difference to perform a strong cleansing (95–95) or a weak cleansing (99–99).

Similarly, a one-way ANOVA was performed for the RF obtaining a *p*-value = 0.00. In this case, the ANOVA indicates that there is strong evidence that the performance differs between the different RF models (when tested against raw unseen data), depending on the cleansing level of the training data.

Once the difference in the performance between the RF models, when tested against raw data is detected *t*-tests were performed in order to identify which models have a different performance. The results of these comparisons are presented in Table 17.

In this case, Table 16 demonstrates that the when comparing models with a similar cleansing level in the second step, the performance is similar. This behavior can be observed in rows 2 and 5 in Table 17. On the other hand, when the cleansing level is different in step 2 the *p*-value is 0.00, meaning that there is a statistically significant difference in the performance of the models. This behavior can be observed in rows 1, 3, 4 and 6 in Table 17.

The last comparison was made to identify if there was a difference in the performance between KNN models when trained with cleaned and raw data; however, tested in both cases against raw data. This means that the F-measure of KNN trained with raw data was compared with KNN trained with cleaned data. In these cases, the performance was calculated testing each model against raw unseen data. These performances can be observed in Table 11. The results of the *t*-test with a significance level of 0.01 can be found in Table 18.

A similar comparison was performed for the RF. The results of the *t*-test with a significance level of 0.01 are presented in Table 19.

## 6. Conclusions

Considering that the aim of the study was to evaluate the impact of the multivariate outlier detection and data cleansing in the performance of different classifiers for HAR. It is important to point out that the cleansing process demonstrated an increase of 12.5% in the F-measure of the KNN classifier. Table 13 presented that the F-measure for the KNN when trained with raw data attained 56%, however, when trained with cleaned data it rises to 63% approximately, in both cases tested against raw unseen data. When performing a *t*-test comparing these two performances, the test yielded a significant difference between the models trained with cleaned data and models trained with raw data. This finding is important considering that the models were tested against raw unseen data. Additionally, it is important to highlight that for KNN, the cleansing levels used in the study have a similar effect in the performance of KNN. This can be concluded as the *p*-value was 0.99 when achieved from the ANOVA analysis within the four KNN cleaned models. 

Other important findings are: 

In the case of RF, the performance dropped when testing the RF cleaned models against raw data. From Table 14, it can be observed that the F-measure for RF when trained with raw data attained 95%; however—when trained with cleaned data—it decreased approximately to 89.5%.

For RF, the cleansing levels used in this study have different effects on the performance of RF. This can be concluded as the *p*-value was 0.00 when performing ANOVA within the four RF cleaned models. In this case, the models with better F-measure against raw data, were the models with the weak cleansing (99%–99%).

The impact of any cleaning process on the classifier should be made against unseen data, and preferably against raw data. In the case of KNN the difference of F-measure attained 30% when tested against cleaned data when compared against raw data. In the case of RF, there is a significant difference too. This difference in the F-measure is close to 5%. 

The reason of the difference in the performance lies on the fact that KNN is a proximity/density classifier and RF is a tree-type classifier. Therefore, it behaves better when trained with raw data because it creates rules for the particular cases (outliers). In the case of KNN, outliers create a distortion in the predictive ability of the classifier. This distortion can be improved with the cleansing process. This situation makes RF the most suitable classifier for HAR, considering that it can better cope with variations in the way activities are being carried out. In this sense, it is also important to highlight that the cleansing process impact is strongly related with the similarity between the classifier and the cleansing process method. In this case, MD has a relation with KNN classifying method in the sense that both are proximity/density methods. However, has no relation at all with random forest. Regarding the performance of both classifiers it is important to point out that according to [63] it is necessary to evaluate the fact that young people is carrying out these tests but the real-world application will be for elderly. This may induce a prediction error.

The features considered for the model can be a mixture of HAR classic features (energy, SMV, among others), and statistical criteria such as exponential, and logarithm. Both were considered significant to improve the predictive ability of both KNN and RF.

For future research, it will be important to check the combinations of models so that a hierarchy could help in the determination of the different classes. In this sense, it would be interesting to consider the combination of random forest with a CNN, similar to what was done by [62] where a hybrid hierarchical classification algorithm (HHC) is presented combining deep learning and threshold-based methods to improve accuracy and rapid computation classifying 15 complex activities. It is also important to verify if the suitable classifiers would change in case that the data are taken from different profile participants (elderly people, mature people, or people with some specifically physical or neurological condition, for example) and how would this would affect the classifier’s performance and the need to include additional features to the process. 

Another important line of investigation would be the inclusion of Lasso regression, LARON (least angle regression outlier nomination) and LARS (least angle regression) to deal with outliers in the process of selecting the best features in order to reduce confusion that outliers can induce to the process of feature selection. This could improve the performance of the models when tested against raw unseen data.

Finally, it would be important to run these models involving also feature selection during the training phase in order to reduce the probability of overfitting.

## Figures and Tables

**Table 1:** Relevant research regarding outlier detection in HAR.

Authors	Outlier Approach	Scope	Relevant Topics
Meng et al. [51]	Heuristic based on statistical hierarchy	Activities of daily living	The model looks for the detection of abnormal behavior once the classifier is trained. Aims to the detect changes in patients’ behavior. No description of data cleaning process.
Gopalakrishnan and Krishnan [52]	Hybrid approach, combining outliers scores	Activities of daily living (ADL)	Improvement of 6% in F-measure, in SVM and RBFN. Database with six activities.
Jones et al. [53]	Hybrid approach using different outlier detection techniques.	Physical activity	Improvement in the performance of the k-means in 4%.
Muñoz-Organero [54]	ANN	ADL	They look for a specific type of outlier data, related with the cessation of movement.
Sunderland et al. [55]	Hybrid approach using simulation and minimum covariance determinant	Database of neurodegenerative disease	They succeeded on the detection of error in the database.
Li et al. [41]	Hybrid approach based on KNN and kernel-target alignment	Not specific	Slight Improvement on the performance of the classifier. Tested in two-class scenarios.
Hyndman and Ullah [50]	Principal component analysis	Forecasting mortality and fertility rates	Reduction of the forecast error.
Penny and Jollyffe [47]	Comparison between different techniques	Laboratory safety data	Concludes that the best approaches for high dimensional data are Mahalanobis distance and Hadi method.
Zhao et al. [56]	Hybrid approach	ADL	Improvement in classifier performance. Tested in data with four activities.
Marubini and Orenti [57]	Minimum covariance determinant	Linear regression prediction	Reduction of the prediction error.

**Table 2:** Scenarios and activities performed [57].

No.	Scenario	Activities	Number of Files
1	Self-care	Hair grooming, washing hands, brushing teeth	24 (72 files)
2	Exercise (cardio)	Walking, jogging, stepping-up.	23 (69 files)
3	House cleaning	Ironing clothes, washing windows, washing dishes	25 (75 files)
4	Exercise (weights)	Arm curls, dead lift, lateral arm raise	21 (63 files)
5	Sport	Bounce ball, catch ball, pass ball	25 (75 files)
6	Food preparation	Mixing food, chopping vegetables, sieving flour	23 (69 files)
Total			141 (423 files)

**Table 3:** Extracted features.

Feature Number	Feature Name	Feature Description
1–3	Mean acceleration	Mean acceleration in *x*, *y*, and *z*, in the time window.
4	Mean *SMV*	Mean *SMV* in the time window. *SMV* was calculated with the relation: $SMV_{i}=\sqrt[{2}]{\left(acc_{x}^{2}+acc_{y}^{2}+acc_{z}^{2}\right)}$ where $acc_{x},\;acc_{y},\;acc_{z}$, denote the acceleration in each axis of the *i*-th observation.
5–7	Mean logarithm	Mean logarithm in x, y and z in the time window.
8–10	Mean exponential	Mean value of the exponential function powered at the value of the acceleration in each axis. This was also calculated in the corresponding time window.
11–13	Mean exponential squared	Mean value of the exponential function powered at the squared value of the acceleration in each axis. This was also calculated in the corresponding time window.
14–16	Mean squared acceleration	Mean of the squared values of the acceleration in x, y and z, in the time window.
17–20	Trapezoidal rule	Trapezoidal rule of acceleration in each axis and *SMV*, in the time window.
21–24	Minimum	Minimum value of the acceleration in each axis, and *SMV* in each time window.
25–28	Maximum	Maximum value of the acceleration in each axis, and *SMV* in each time window.
29–32	Range	Range of the values of acceleration in each axis and *SMV* in the time window.
33–36	Standard deviation	Standard deviation of the values of *SMV* and acceleration in the three axis, in the time window.
37–40	Root mean square (*RMS*)	Root mean square of the values of *SMV*, and acceleration in each axis, in the time window. RMS is calculated according to the relation $RMS_{x}=\sqrt[{2}]{mean\left(acc_{x}^{2}\right)}$
41	Signal magnitude area (*SMA*)	Signal magnitude area in the time window. It is calculated with the equation $SMA=\overset{n}{\underset{i=1}{\sum }}\left(\left|acc_{x\;i}\right|+\left|acc_{y\;i}\right|+\left|acc_{z\;i}\right|\right)$ where, $\left|acc_{x\;i}\right|$ corresponds to the absolute value of the i-th observation of the acceleration across *x*-axis in the time window.
42–44	Mean square	Mean of the square values of individual observations in the time window.
45–48	Entropy	Fast Fourier transform of *SMV*, and across *x*, *y*, and *z*-axis, in the time window.
49–51	Median	Median of the acceleration values across *x*, *y*, and *z*-axis in the time window.

**Table 4:** Distribution of the data by activity.

Activity	No. of Files
Arm curls	21
Bounce	25
Catch	25
Chopping	23
Deadlift	21
Dishwashing	25
Hair grooming	25
Handwashing	24
Ironing	24
Jogging	23
Lateral arm raise	21
Mixing food in bowl	23
Pass	25
Sieving flour	23
Stepping	23
Teeth brushing	24
Walking	23
Window washing	25
TOTAL	423

**Table 5:** F-measure results of the different classifiers at stage 1.

Classifier	F-Measure
KNN	84.00%
SVM	73.60%
Random forest	89.40%
Neural networks	78.00%
Regression	72.10%

**Table 6:** F-measure value with different cleansing levels.

F-Measure			
Scenario	KNN	RF	Kept Instances (%)
Case 1	95.80%	96.00%	84.07%
Case 2	94.70%	95.70%	92.99%
Case 3	94.10%	93.80%	88.52%
Case 4	81.20%	93.90%	98.16%
Case 5	52.20%	92.10%	100%

**Table 7:** F-measure value with different cleansing levels and different amount of features.

Scenario	KNN (27 Features)	KNN (3 Features)	Kept Instances
Scenario 1	92.90%	63.80%	98.5%
Scenario 2	94.20%	64.00%	88.7%

**Table 8:** F-measure value with different cleansing levels for KNN against the corresponding cleaned test set.

KNN	Cleaning Levels			
Fold	95_95	95_99	99_95	99_99
**1**	0.940	0.925	0.938	0.921
**2**	0.935	0.922	0.929	0.914
**3**	0.935	0.923	0.936	0.922
**4**	0.939	0.867	0.930	0.860
**5**	0.941	0.936	0.936	0.842
**6**	0.949	0.842	0.936	0.855
**7**	0.935	0.860	0.932	0.838
**8**	0.936	0.866	0.934	0.833
**9**	0.937	0.926	0.938	0.924
**10**	0.933	0.849	0.933	0.846

**Table 9:** F-measure value with different cleansing levels for RF against the corresponding cleaned test set.

RF	95_95	95_99	99_95	99_99
**1**	0.953	0.948	0.946	0.946
**2**	0.952	0.948	0.949	0.944
**3**	0.952	0.951	0.949	0.946
**4**	0.952	0.948	0.947	0.946
**5**	0.950	0.951	0.948	0.944
**6**	0.954	0.946	0.946	0.946
**7**	0.950	0.948	0.942	0.947
**8**	0.950	0.951	0.948	0.945
**9**	0.952	0.949	0.946	0.944
**10**	0.953	0.946	0.945	0.943

**Table 10:** F-measure value for KNN and RF with raw data. Trained and tested against raw data.

	RF	KNN
**1**	0.939	0.557
**2**	0.942	0.556
**3**	0.945	0.555
**4**	0.941	0.562
**5**	0.941	0.561
**6**	0.937	0.549
**7**	0.938	0.543
**8**	0.941	0.551
**9**	0.94	0.559
**10**	0.94	0.557

**Table 11:** F-measure value for each KNN and RF model when tested against raw data.

Repetition	Models									
	KNN raw	RF raw	95_95 KNN	95_99 KNN	99_95 KNN	99_99 KNN	95_95 RF	95_99 RF	99_95 RF	99_99 RF
**1**	55.91%	94.35%	55.61%	56.34%	55.63%	56.17%	88.15%	90.80%	88.34%	90.92%
**2**	55.04%	94.47%	73.13%	75.26%	73.01%	75.38%	88.2%	90.30%	87.80%	90.73%
**3**	55.72%	94.09%	59.51%	60.17%	59.06%	60.31%	87.54%	90.14%	87.35%	90.47%
**4**	55.56%	94.44%	63.39%	62.56%	63.43%	62.65%	87.51%	90.71%	87.91%	91.30%
**5**	56.67%	94.73%	57.43%	57.99%	57.40%	58.07%	87.73%	90.87%	88.03%	89.97%
**6**	56.27%	94.61%	69.16%	66.34%	69.51%	66.86%	87.89%	90.82%	88.17%	91.75%
**7**	57.05%	95.15%	63.86%	61.28%	64.71%	61.24%	88.91%	91.56%	88.58%	92.05%
**8**	55.56%	94.44%	68.00%	69.09%	68.28%	69.11%	88.10%	90.42%	88.48%	91.23%
**9**	56.17%	94.47%	64.69%	65.45%	65.28%	65.71%	87.63%	90.78%	87.80%	91.11%
**10**	55.87%	95.1%	59.67%	59.67%	59.56%	59.44%	88.08%	91.11%	88.55%	90.78%

**Table 12:** Mean, standard deviation, and *p*-value for Anderson Darling test, for the KNN models tested against cleaned sets.

	95_95 KNN	95_99 KNN	99_95 KNN	99_99 KNN
Mean	93.8%	89.2%	93.4%	87.6%
Standard Deviation	0.46%	3.76%	0.32%	3.93%
*P*-Value	0.0763	0.0189	0.4524	0.0128

**Table 13:** Mean, standard deviation, and *p*-value for Anderson Darling test, for the RF models tested against cleaned sets.

	95_95 KNN	95_99 KNN	99_95 KNN	99_99 KNN
Mean	95.2%	94.9%	94.7%	94.5%
Standard Deviation	0.14%	0.19%	0.21%	0.13%
*P*-Value	0.083	0.0789	0.0.3061	0.1096

**Table 14:** Mean, standard deviation, and *p*-value for Anderson Darling test, for the RF and KNN models tested against raw sets.

	KNN raw	RF raw	95_95 KNN	95_99 KNN	99_95 KNN	99_99 KNN	95_95 RF	95_99 RF	99_95 RF	99_99 RF
Mean	55.98%	94.58%	63.40%	63.42%	63.59%	63.49%	87.97%	90.75%	88.10%	91.03%
Standard Deviation	0.58%	0.33%	5.59%	5.72%	5.66%	5.82%	0.42%	0.41%	0.40%	0.60%
*P*-Value	0.8347	0.1165	0.8579	0.5615	0.8644	0.6167	0.2539	0.5110	0.6290	0.9490

**Table 15:** *p*-value for *t*-test comparison for KNN models (tested against cleaned data).

Model 1	Model 2	*p*-Value
95_95_KNN	95_99_ KNN	0.003
95_95_ KNN	99_95 _ KNN	0.047
95_95_ KNN	99_99_ KNN	0.0007
95_99_ KNN	99_95 _ KNN	0.006
95_99_ KNN	99_99_ KNN	0.361
99_95 _ KNN	99_99_ KNN	0.001

**Table 16:** *p*-value for *t*-test comparison for the RF models (tested against cleaned data).

Model 1	Model 2	*p*-Value
95_95_RF	95_99_ RF	0.0
95_95_ RF	99_95 _ RF	0.0
95_95_ RF	99_99_ RF	0.0
95_99_ RF	99_95 _ RF	0.039
95_99_ RF	99_99_ RF	0.0
99_95 _ RF	99_99_ RF	0.07

**Table 17:** *p*-value for *t*-test comparison the for RF models (tested against raw data).

Model 1	Model 2	*p*-Value
95_95_RF	95_99_ RF	0.000
95_95_RF	99_95 _RF	0.494
95_95_RF	99_99_RF	0.000
95_99_ RF	99_95 _RF	0.000
95_99_ RF	99_99_RF	0.243
99_95 _RF	99_99_RF	0.000

**Table 18:** *p*-value for *t*-test comparison for the KNN (raw data model) against KNN (cleaned data models) when tested against raw data.

Raw Data Model	Clean Data Model	*p*-Value
KNN	95_95_KNN	0.002
KNN	95_99_ KNN	0.003
KNN	99_95 _KNN	0.002
KNN	99_99_KNN	0.003

**Table 19:** *p*-value for *t*-test comparison for the RF (raw data model) against RF (cleaned data models) when tested against raw data.

Raw Data Model	Clean Data Model	*p*-Value
RF	95_95_RF	0.000
RF	95_99_ RF	0.000
RF	99_95 _RF	0.000
RF	99_99_RF	0.000
